# Olbert’s Balloon Dilatation as a Minimally Invasive Possibility of Treating Ureteral Stricture After Complicated URS-L in Children

**DOI:** 10.3389/fped.2022.767500

**Published:** 2022-08-29

**Authors:** Adam Halinski, Andrzej Halinski, Paweł Halinski

**Affiliations:** ^1^Department of Pediatric Urology, Private Medical Center “Klinika Wisniowa”, Zielona Góra, Poland; ^2^Department of Clinical Genetics and Pathology, University of Zielona Gora, Zielona Gora, Poland; ^3^Scientific Office, U-merge Ltd., London-Athens-Dubai, Athens, Greece

**Keywords:** ureterorenoscopy, lithotripsy, Olbert balloon, dilatation, JJ stent

## Abstract

Olbert’s balloon dilatation is a surgical technique used for the treatment of ureteral stricture. Although it is more frequently used in adults, due to the advancing miniaturization of the equipment, this technique has become possible in children. We would like to present five cases of Olbert’s balloon dilatation carried out in children with ureteral stricture, aged 12–17 years. All of these children were diagnosed for at least 6 months. Ureteral stricture has been noticed in those patients with a time of a stone residence in the ureter longer than 6 months. The duration of the stone in the ureter varied from 6 to 18 months. The symptoms were abdominal pain, renal colic pain, UTI, fever, vomiting, and nausea. Ultrasound (US) revealed hydronephrosis and ureter dilatation above the stone. All of these children had grade-3 hydronephrosis based on the Onen grading system during admission to the hospital. The lack of renal function on the DMSA scan was observed with an average of 22%. JJ-stent was inserted as a first-line treatment. A retrograde pyelogram revealed ureteral stricture at a length from 1 to 1.7 cm in the place where the stone was ingrown. Olbert’s balloon dilatation under fluoroscopy was performed successfully in all children. We achieved an efficacy of 60% in our series. Renal function increased to an average of 36% on DMSA 3 months after surgery. The level of creatinine is shaped at an average of 0.6. On US, two children had no hydronephrosis while one child had grade-1 hydronephrosis. The longest follow-up is now 4 years, with the same good results. In conclusion, Olbert’s balloon dilatation is an effective, safe, and minimally invasive tool for ureteral stricture in the hands of the endourological experienced pediatric urologist. But more prospective, randomized trials are still needed.

## Introduction

Olbert’s balloon dilatation is a surgical technique used for the treatment of ureteral stricture. It is more frequently used in adults (1, 2). Due to the advancing miniaturization of the equipment and its precision, this technique has become possible in the treatment process in children.

Olbert balloon is manufactured and patented (EP0186267A1) by COOK Medical balloon catheter having an expandable and collapsible elastic balloon is shown, wherein the balloon is reinforced by knitted fabric such that the balloon cannot expand beyond a predetermined diameter regardless of the internal pressure applied to the balloon. The knitted construction permits the balloon to expand in diameter without shortening in length and permits the balloon to collapse smoothly without folds and wrinkles (3).

The aim of this publication is to present the possibilities of treating ureteral stricture with the use of an Olbert balloon.

## Materials and Methods

We would like to present five cases of Olbert’s balloon dilatation carried out in children with ureteral stricture after complicated URS-L. The children were treated from January 2016 to February 2019. Ureteral stricture has been noticed in those patients with a time of a stone residence longer than 6 months in the ureter. The manuscript as a retrospective study was accepted by the Collegium Medicum University of Zielona Gora Ethical Committee.

**FIGURE 1:**
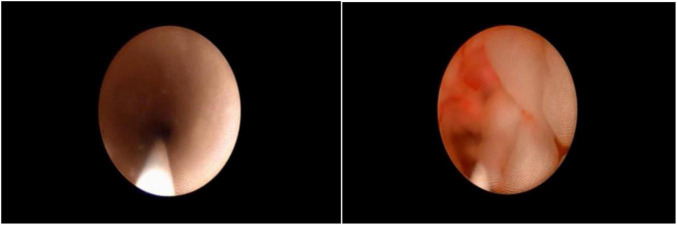
Ureterorenoscopy procedure. Edema of the ureteral wall.

All of these children were diagnosed for at least 6 months. The duration of the stone in the ureter varied from 6 to 18 months. The symptoms were: abdominal pain, renal colic, UTI, fever, vomiting, and nausea. Ureterorenoscopy was performed. Because of the edema of the ureteral wall (Figure 1) only JJ-stent was inserted as a first-line treatment. As a second procedure, we performed URS-L. During the lithotripsy stone ingrown in the ureteral wall was found and a 4.7 Fr JJ-stent was left for 3 months after the surgery. The children were observed in the out-patient clinic every 4 weeks without any complaints, UTI, and hydronephrosis during ultrasound check-up. We assessed hydronephrosis on ultrasound based on the Onen hydronephrosis grading system (4).

**FIGURE 2:**
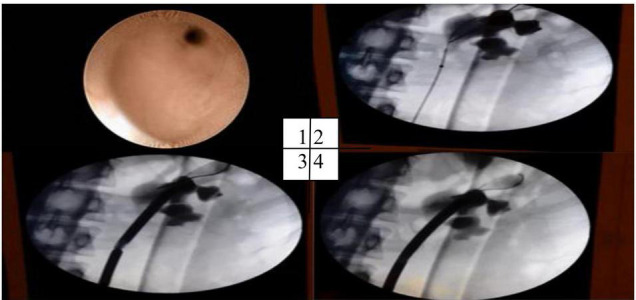
Olbert’s balloon dilatation. Endoscopy view of the stricture from below. (1) Insertion of the Olbert balloon, (2) stricture during, (3) and after dilatation (4).

For our surgery, we used a balloon with a length of 6 or 10 cm and a diameter of 6 mm each, manufactured by COOK Medical. Retrograde pyelography was first performed to visualize the place of a stricture. All balloons were inserted on guide wire under fluoroscopy control. The distal marker was placed above and proximal under the stricture. The balloon was then inflated with contrast media solution to control the stage of the procedure and to evaluate ureteral stenosis (Figure 2).

## Results

The average age was 14 (range 12–17) years. Ultrasound (US) revealed hydronephrosis and ureter dilatation above the stone. All of these children had grade-3 hydronephrosis based on the Onen grading system during admission to the hospital. The lack of renal function on the DMSA scan was observed with an average of 22% before treatment (Table 1).

**TABLE 1:** Patient series (age, length of stenosis, DMSA, degree of hydronephrosis before and after surgery, and follow-up).

	Patient age	Stricture length (cm)	DMSA before the surgery	DMSA after the surgery	Grade of hydronephrosis before the surgery	Grade of hydronephrosis after the surgery	Follow up
Case 1	12	1	20	39	3-rd grade	No hydronephrosis	2 years
Case 2	14	1,2	22	44	3-rd grade	No hydronephrosis	4 years
Case 3	15	1,5	21	28	3-rd grade	1-st grade hydronephrosis	3 years
Case 4	17	1	21	26	3-rd grade	3-rd grade hydronephrosis	Ureter reimplantation
Case 5	12	1,7	26	43	3-rd grade	No hydronephrosis	4 years

All children tolerated the DJ catheter very well. After 3 months stent was removed. Abdominal and/or renal side pain and UTI developed after removing the JJ-stent. Ultrasound examination showed progressive urine retention in the kidney, without visualization of the stone in the ureter.

A second URS procedure was performed. A retrograde pyelogram revealed ureteral stricture at a length of 1–1.7 cm in the place where the stone was ingrown in the ureteral wall. Olbert’s balloon dilatation under fluoroscopy was performed successfully (Figure 2).

During the URS-L procedure in all children, large edema of the ureteral wall and the ingrown to the ureteral wall stone were noticed. The stone-free rate was 100% after URS-L.

The level of creatinine is shaped at an average of 0.6. Renal function increased to an average of 36% on DMSA 3 months after surgery.

We achieved an efficacy of 60% in our series, after Olbert’s balloon dilatation under fluoroscopy. On US, three children had no hydronephrosis while one child had grade-1 hydronephrosis. One child required a ureter resection and anastomosis due to severe hydronephrosis and persistent ureteral stricture after balloon dilatation. No major intraoperative or postoperative complications were noticed. Persistent hematuria remains till 3rd day. No symptomatic UTI was observed, due to antibiotic prophylaxis, which was begun 3 days before the surgery, according to the urine culture and antibiogram. The longest follow-up in the out-patient clinic is now 4 years, with the same good results.

## Discussion

In the pediatric population, the ureteral stricture is mainly a congenital condition. Therefore, ureteral trauma is a rare condition [1–2.5% urinary tract trauma (5)]. What is more, it is frequently missed intraoperatively (6). The causes of the ureteral strictures can be divided into two groups. The most common reason is iatrogenic one, which is responsible for 98% of cases consisting of endourology, open and laparoscopic abdominal or pelvic surgeries, and radiotherapy (7). The rest 2% are external factors including blunt or penetrating injuries (5). There are several other factors that may increase the likelihood of ureteral damage, such as: birth defects, tumors, skeletal system deformities, postoperative conditions, radiotherapy, and the long presence of stones in the ureter (7). The number of urological iatrogenic trauma has decreased in the last years, due to improvements in surgical experience, techniques, and instruments (8). Traxer has systematized and divided ureteral injuries into 5 degrees: type 0—no injury; type 1—shallow mucosal damage; type 2—deep mucosal damage; type 3—perforation of the ureter; and type 4—avulsion of the ureter (9). Symptoms of ureter damage are non-specific, depending on age, type, and degree of damage. They include lumbar or lower abdominal pain, fever, hematuria, dysuric symptoms, and reduction in diuresis (10). We have to remember that in about 25% of cases, iatrogenic ureteral damage may initially be asymptomatic and manifest after a few weeks as a fistula.

Diagnostic tools include assessment of the patient’s condition and laboratory tests (biochemical and urine tests). In contrast to adults, erythrocyturia is not observed in more than 50% of injuries in children. Computed tomography of the abdomen with an intravenous contrast medium is the diagnostic examination of choice (11). It is important to obtain late images about 15–20 min after administration of the contrast media, to improve diagnosis and show ureteral extravasation.

Management of ureteral trauma differs depending on many factors and could be a multistep procedure. Partial, late-diagnosed partial ureteral injuries can be managed first with a JJ-stent or nephrostomy tube placement with the a success rate of 14–19% (7, 12, 13). However, due to the EAU guidelines, endourological treatment of small ureteral strictures is feasible, safe, and effective (7). In case of failure, an open or laparoscopic surgical repair is necessary.

According to the data in the literature, Rivas et al. prove that high-pressure balloon dilatation is safe and effective in ureteral stricture after reconstructive surgery (14). Conclusions from the study on long-term outcomes in Primary Obstructive Megaureter treated with balloon dilatation from high volume center Hospital General Universitario Gregorio Maranon Madrid, Spain have shown to be an effective treatment with few complications and good results at long-term follow-up (15). As we know ureteral strictures can affect both proximal and distal ureter in the literature are several manuscripts concerning balloon dilatation in Ureteropelvic Junction Obstruction (UPJ) as a minimally invasive and effective treatment (16). Alberto Parente et al. showed that there is a place for balloon dilatation in recurrent UPJ obstruction (17). However, while browsing the available databases, we found no publications on high-pressure balloons in ureteral strictures after ureterorenoscopy—lithotripsy procedures. We, as authors, believe that this publication may be the beginning of creating indications for the treatment of ureteral stricture after complicated URS-L surgery. The limitations of this manuscript are: the number of cases and retrospective analysis. In the future prospective, randomized trials are needed.

## Conclusion

Olbert’s balloon dilatation is an effective, safe, and minimally invasive tool for ureteral stricture in the hands of the endourological experienced pediatric urologist. We believe that the advancing miniaturization of the equipment and gaining experience will enable carrying out of this procedure even in smaller children with high efficiency. We also believe that children will be better and faster diagnosed and referred to a pediatric urology department with a high volume of URS-L procedure at once.

## Data Availability Statement

The original contributions presented in this study are included in the article/supplementary material, further inquiries can be directed to the corresponding author.

## Ethics Statement

The studies involving human participants were reviewed and approved by Bioethical Committee at Collegium Medicum, University of Zielona Gora, Poland. Written informed consent to participate in this study was provided by the participants’ legal guardian/next of kin.

## Author Contributions

All authors listed have made a substantial, direct, and intellectual contribution to the work, and approved it for publication.

## Conflict of Interest

AdH was employed by U-merge Ltd. The remaining authors declare that the research was conducted in the absence of any commercial or financial relationships that could be construed as a potential conflict of interest.

## Publisher’s Note

All claims expressed in this article are solely those of the authors and do not necessarily represent those of their affiliated organizations, or those of the publisher, the editors and the reviewers. Any product that may be evaluated in this article, or claim that may be made by its manufacturer, is not guaranteed or endorsed by the publisher.
